# Cytocompatibility of the selected calcium phosphate based bone cements: comparative study in human cell culture

**DOI:** 10.1007/s10856-015-5589-x

**Published:** 2015-10-28

**Authors:** Radosław Olkowski, Piotr Kaszczewski, Joanna Czechowska, Dominika Siek, Dawid Pijocha, Aneta Zima, Anna Ślósarczyk, Małgorzata Lewandowska-Szumieł

**Affiliations:** Department of Histology and Embryology, Centre for Biostructure Research, Medical University of Warsaw, Warsaw, Poland; Department of Pathology, Medical University of Warsaw, Warsaw, Poland; Centre for Preclinical Research and Technology, Medical University of Warsaw, Warsaw, Poland; Faculty of Materials Science and Ceramics, AGH-University of Science and Technology, Kraków, Poland

## Abstract

Calcium phosphate cements (CPC) are valuable bone fillers. Recently they have been also considered as the basis for drug-, growth factors- or cells-delivery systems. Broad possibilities to manipulate CPC composition provide a unique opportunity to obtain materials with a wide range of physicochemical properties. In this study we show that CPC composition significantly influences cell response. Human bone derived cells were exposed to the several well-characterized different cements based on calcium phosphates, magnesium phosphates and calcium sulfate hemihydrate (CSH). Cell viability assays, live/dead staining and real-time observation of cells in contact with the materials (time-laps) were performed. Although all the investigated materials have successfully passed a standard cytocompatibility assay, cell behavior in a direct contact with the materials varied depending on the material and the experimental system. The most recommended were the α-TCP-based materials which proved suitable as a support for cells in a direct contact. The materials which caused a decrease of calcium ions concentration in culture induced the negative cell response, however this effect might be expected efficiently compensated in vivo. All the materials consisting of CSH had negative impact on the cells. The obtained results strongly support running series of cytocompatibility studies for preclinical evaluation of bone cements.

## Introduction

Calcium phosphate cements (CPC) are well recognized biomaterials, widely used in bone substitution due to their chemical and structural similarity to the inorganic component of bone [1–4]. CPC belong to the group of bioactive chemically bonded materials which are able to form a direct connection with bone tissue [2, 5]. Moreover, due to good plasticity these materials are capable of forming thigh filling of existing voids and thus serving as the suitable bone fillers. [6, 7]. Recently they have been also considered as potential scaffolds for bone tissue engineering and taken into account for drug or growth factors delivery systems [8–10]. Following the classical pathway typical for CPC all the materials are composed of two phases: solid and liquid, which upon mixing form a moldable paste that should be applied into the defect area before solidifying [3, 4]. As a solid phase, hydroxyapatite (HA, Ca_10_(PO_4_)_6_(OH)_2_) and α-tricalcium phosphate (α-TCP, α-Ca_3_(PO_4_)_2_) are of particular interest as the components of CPC [11, 12]. Synthetic hydroxyapatite is widely used in clinical practice, due to its excellent biocompatibility and similarity to the inorganic constituent of the mineral part of bone. In some cases, in CPC incorporation of magnesium and carbonate ions into the structure of hydroxyapatite is used, since it positively influences physicochemical and biological properties of cements [13, 14]. The main reason for the growing interest in α-TCP are its setting ability and biodegradability. α-TCP based biomaterials are more resorbable than HA, β-TCP and biphasic (HA, β-TCP) bioceramics currently available on the market [11]. Furthermore, α-TCP is able to transform into hydroxyapatite under physiological and close to physiological conditions [11, 12]. Most of the published research regard α-TCP obtained in high-temperature solid state reaction, however as an alternative, low-temperature process, i.e. the wet chemical synthesis may also be applied successfully [11, 12].

Distilled water and the aqueous solutions of the substances, such as sodium phosphate, citric acid, gelling polymers such as sodium hyaluronate, sodium alginate, chondroitin sulfate, chitosan and methylcellulose may be used as a liquid phase. Application of gelling agents has beneficial effect on consistency of the cement pastes owing to significant improvement of its surgical handling and cohesion in a body fluid environment [15–17].

Another group of inorganic cements, based on magnesium oxide and phosphate compounds, comprises magnesium phosphate cements (MPC). The ongoing studies have shown the advantages of MPC, such as high initial mechanical strength, fast setting and good adhesive properties which make MPC promising materials for bone tissue engineering [18, 19]. Although their biocompatibility has been confirmed [20], mineral phases other than struvite, which may be formed during the setting process of MPC, are described as slightly less biocompatible [21]. Heat release during the setting process of MPC has been the main problem in their development. Temperatures as high as 90 °C in the process of MPC setting have been reported [22]. The release of heat can be significantly reduced by addition of certain substances such as sodium pyrophosphate or sodium borate [18, 22]. Moreover, the use of excess amount of MgO in the initial MPC formulations results in pH increase in the vicinity of the implant material, due to formation of Mg(OH)_2_. Elevated pH value has been found to be toxic to bacteria but it may also be harmful for bone cells [18]. Nevertheless, in contrast to the results obtained by other authors, among the phases present in our composite no unreacted magnesium oxide was detected [23].

Another component of CPC, calcium sulfate hemihydrate (CSH), has a long clinical history in filling bone defects. CSH is known for its biocompatibility, osteoconductivity and fast resorption [24, 25]. The material is surgically manageable, easily accessible and inexpensive and may be an attractive component of cements due to its haemostatic and angiogenic properties [26–28]. Presence of CSH in the solid phase of the cements is one of the main factors influencing this material’s behavior in in vitro environment. The combination of calcium phosphates with CSH allows to produce biphasic composites with shorter setting time and controlled resorption rate [29, 30]. Furthermore, CSH as well as sodium pyrophosphate may be used to delay and control setting reaction of magnesium phosphate-based cements.

The broad range of CPC applications stimulate their continuous development. Vast possibilities of CPC modifications result in a wide variety of the final products, different in mechanical properties, reactivity in biological systems and biocompatibility.

Each detail of the composition, technology and the final form of the material may significantly influence its properties and in a consequence, its suitability for the particular clinical application. Therefore, extensive preclinical examination is required. Observations based on experimental implantation to animal tissues are costly and time consuming. At the same time, due to the interspecies differences and the lack of fully satisfied experimental model, transfer of the data obtained in animals to humans is very often confusing and reliable only to a limited extent. Therefore cytocompatibility studies in cell culture seem invaluable especially in screening tests. On the other hand, although the in vitro experimental systems are far better controlled than the in vivo ones, they cannot be applied in a routine way when the investigated materials are unstable in a biological environment. In the case of modern CPC, the material’s activity in contact with cells and tissues is clinically desirable, but challenging in terms of preclinical investigations. Particularly, ions release or uptake change the composition of the culture medium resulting in different culture conditions.

The aim of this work was to evaluate cytocompatibility of the several different cement type materials based on calcium phosphates, magnesium phosphates and CSH. Their compositions were chosen in accordance with the current trends in CPC technology aiming to improve their physicochemical properties and stability of the final products [31]. The purpose of the study was to observe their behavior under the specific conditions of the cell culture systems in order to evaluate the selected cements. We also aimed at establishing a reliable protocol of cytocompatibility studies—based on a combination of various experimental systems—dedicated to chemically unstable materials. Such observations might serve as a screening assay for the CPC. Additionally, we have tested the materials as support for cell transplantation in advanced therapy medicinal products (ATMP).

## Materials and methods

### Synthesis of the composite cements

The phase compositions of investigated materials, marked as: R1, R2, A1, A2, B1, C1 and C2 are presented in Table 1. All studied materials consisted of the solid and liquid phase. Materials R1 and R2 were commercially available bone substitutes, known as HydroSet™ (Stryker^®^) and Surgi Plaster™ P30 (GHIMAS^®^), respectively and were used as reference materials. All the other materials were developed and prepared at the AGH University of Science and Technology in Krakow (Poland). Materials A1 and A2 were manufactured from powder composed of magnesium phosphate cement (MPC), hydroxyapatite (HA) and CSH, (all from Across Organics, USA) or sodium pyrophosphate (Na_4_P_2_O_7_·10H_2_O, POCH, Poland). Magnesium phosphate cement was obtained by mixing NH_4_H_2_PO_4_ (Chempur, Poland) and MgO in equimolar proportions. MgO was obtained by calcination of 4MgCO·Mg(OH)_2_·5H_2_O (POCH, Poland) at the temperature above 1100 °C. Hydroxyapatite was synthesized by the wet method from calcium oxide (POCH, Poland) and phosphoric acid (POCH, Poland). Initial B1 powder was prepared by mixing magnesium doped carbonated hydroxyapatite (MgCHA) with CSH (Acros Organics, USA). MgCHA was produced by the wet method using as starting materials Ca(OH)_2_ (Merck, Poland), (NH_4_)_2_HPO_4_ (POCH, Poland), (CH_3_COO)_2_Mg (POCH, Poland) and NH_4_HCO_3_ (POCH, Poland). Synthesized MgCHA powder was calcined at 400 °C. Solid phase of materials C1 and C2 consisted of α-tricalcium phosphate (α-TCP). α-TCP powder was obtained by the wet chemical method. Ca(OH)_2_ (POCH, Poland) and 85 wt% H_3_PO_4_ solution (POCH, Poland) were used as the substrates. The resulting precipitates were aged, dried, sintered, milled in attritor and sieved (mesh size 0.063 mm). Various liquid phases were used to produce cement pastes, i.e.: distilled water for A1 and A2, 1.0 wt% chitosan solution in 0.3 wt% acetic acid (Sigma-Aldrich, Germany) for B1 and 1.0 wt% chitosan solution in 0.5 wt% acetic acid (Sigma-Aldrich, Germany) or 0.75 wt% methylcellulose solution in 2.0 wt% Na_2_HPO_4_ for C1 and C2, respectively (Table 1).

**Table 1:** Solid and liquid phases of developed biomaterials

Biomaterial	Solid phase	Liquid phase	L/P (g/g)
R1 (I reference material)	DCPD (*dicalcium phosphate dihydrate*) TTCP (*tetracalcium phosphate*), Tri-sodium citrate [18]	Sodium phosphate, *polyvinylpyrrolidone* water [18]	0.33
R2 (II reference material)	CSH (100 wt%) [19]	REGULAR liquid [19]	0.50
A1	HA (46 wt%), MPC (46 wt%), sodium pyrophosphate (8 wt%)	distilled water	0.40
A2	HA (40 wt%), MPC (35 wt%), CSH (15 wt%),	distilled water	0.48
B1	MgCHA (40 wt%), CSH (60 wt%),	1.0 wt% chitosan solution in 0.3 wt% acetic acid	0.54
C1	α-TCP (100 wt%),	1.0 wt% chitosan solution in 0.5 wt% acetic acid	0.48
C2	α-TCP (100 wt%),	0.75 wt% methylcellulose solution in 2.0 wt% Na_2_HPO_4_	0.48

Cement specimens for in vitro tests were prepared by mixing the appropriate amounts of the solid and liquid phase to produce easily moldable paste. Afterwards, the paste was placed into the Teflon mold (4 mm in height, 6 mm in diameter) and left to set. Samples were sterilized with 25 kGy radiation.

### Characteristics of the cements

Phase composition of all the developed biomaterials was determined by X-ray method. Measurements were carried out by X-ray diffractometer *X’Pert Pro* (Philips) using CuKα radiation within the 2θ range from 10° to 90° at a scanning speed of 10° min^−1^. In order to perform quantitative analysis of phase composition of set and hardened cement bodies the Rietveld method was used.

Mercury intrusion porosimetry (MIP) was applied to examine the open porosity and pore size distribution in the examined materials. The measurements were carried out using porosimeter AutoPore IV (Micromeritics).

### Cell culture

Human bone-derived cells (hBDC) were used for cytocompatibility tests. Cells were isolated from pieces of bone explanted postsurgery. All the procedures were approved by the Local Ethics Committee of the Medical University of Warsaw (Decision No. KB/74/2005) and the donors provided the informed consent. hBDC used for experiment were isolated from femurs or ribs of 3 donors: two females and one male; age 33, 58 and 67 years. The isolation was based on the protocols described by Gallagher et al. [32] with modifications [33]. Briefly, bone chips obtained from surgery were cleaned of the connective tissue, cut into 1–2 mm fragments, rinsed with PBS (Life Technologies), and incubated overnight in medium containing collagenase (Sigma) at 37 °C. After incubation, bone fragments were rinsed in PBS to remove the remains of soft tissue and cultured in vitro in Dulbecco’s Modified Eagle Medium (DMEM), supplemented with 10 % Foetal Bovine Serum (FBS), 1 % l-glutamine, 1 % Antibiotic–Antimycotic (all media from Life Technologies), ascorbic acid (30 μg/ml; Sigma-Aldrich). Cells expanded from the bone chips were seeded onto the ceramic samples or into wells of a culture plate. Cells from different donors were never pooled; in each experiment cells from only one donor were used. hBDC from different donors were used for subsequent experiment repetitions.

Assessment of cytocompatibility in vitro was performed by cell metabolic activity measurement, fluorescent staining and microscopic analysis of cell morphology.

### Cell viability assays

Metabolic activity measurements were performed with two assays: XTT (Sigma-Aldrich) [34] and Alamar Blue (Life Technologies) [35]. Both methods are based on redox activity of living cells. In XTT assay, water soluble tetrazolium salt (XTT) is reduced to chromogenic formazan by cellular succinate dehydrogenase. In Alamar Blue assay blue, non-fluorescent resazurin is reduced to red, fluorescent resorufin. Metabolic activity was assessed by absorbance (XTT) or fluorescence (Alamar Blue) measurement of reaction products read in the ELISA reader (FLUOstar OPTIMA, Germany). The results were proportional to the number of living cells.

In order to visualize live and dead cells Live/Dead kit (Life Technologies) was used. Calcein acetoxymethyl ester was converted into fluorescent calcein only by the living cells, while ethidium homodimer-1 stained dead cells only. In fluorescent microscope living cells were green, while dead cells were red [36].

The results of cell viability assays were presented as mean ± SD. To evaluate the significance of the differences Kruskal–Wallis non-parametric ANOVA with following post hoc test was performed using STATISTICA 10 (StatSoft, Tulsa, OK, USA). For statistical analyses, *P* < 0.05 was considered statistically significant.

### Cell imaging

Microscopic phase contrast and fluorescence observations were carried out with Nikon Eclipse TE2000-U inverted microscope with NIS-Elements F software.

Real-time microscopic observation of cell culture was carried out for 36 h in Nikon Eclipse Ti microscope with incubator. NIS-Elements BR software was used to time-lapse image capturing.

### Cytotoxicity assessment with the use of the extracts

Extracts from ceramic materials were prepared according to ISO 10993-12 [37]. Extraction of ceramic samples was carried out for 24 h in DMEM (Invitrogen). Volume/mass proportion was 1 ml of DMEM per 0.05 g of ceramic material. According to ISO 10993-12 guidelines, negative control (substance which demonstrates non-reactive response in the test system) as well as positive control (substance which demonstrates reproducible, cytotoxic response) were prepared. Alumina samples were used as negative control and 0.1 % solution of Triton X-100 (Sigma) as positive control. Cells were cultured in standard culture medium.

In vitro cytotoxicity evaluation was assayed according to ISO 10993-5 [38]. hBDC were seeded in 96-well culture plate (1.5 × 10^4^ cells per well). On the next day, the medium was replaced by either 100 % extracts or 50 % extracts from the materials in DMEM, always supplemented with FBS, l-glutamine and Antibiotic–Antimycotic solution (to the final concentration of 10, 1 and 1 %, respectively). After 24 h culture microscopic observation of cells was performed and cellular metabolic activity was measured with XTT assay. The observation was performed in three independent experiments. According to ISO 10993-5, a decrease in metabolic activity below 70 % of control (hBDC cultured in standard medium) was considered as cytotoxic effect.

Concentration of calcium ions in extracts was measured by Calcium Colorimetric Assay Kit (BioVision). Calcium present in extract formed chromogenic complex with *o*-cresophtalein and calcium concentration was evaluated by absorbance reading.

### Observations in a direct contact of cells with solid composite samples

Ceramic samples used for tests in direct contact with cells were incubated in calcium/magnesium-free PBS for 12 h. They were then placed into 24-well untreated plate and seeded with 5000 hBDC per sample in 1 ml of culture medium. Cells growing in tissue culture-treated polystyrene (TCPS) culture plate served as a control. After 48 h, cell viability was estimated by Alamar Blue test and by Live/Dead fluorescent staining with following microscopic observation. The observation was carried out in three independent experiments.

The ceramic materials showing the highest cytocompatibility in 24-well plate underwent further tests. The samples were incubated for 12 h in sterile, distilled water, then inserted into wells of 96-well plate and seeded with 1.5 × 10^4^ cells in 0.2 ml of culture medium. In this experimental system ceramic samples covered the whole area accessible for cell adhesion. After 48 h of cell culture, cell viability was estimated by Alamar Blue test. It is worth emphasizing that due to the different volume of cell culture medium in 24- and 96- well plates the effect of the tested insert on medium composition might differ.

In order to estimate cytotoxicity of ceramic materials in indirect contact with cells, samples were placed in 24-well culture plates, which were previously seeded with hBDC. In vitro cell culture was continued for 36 h. Real-time microscopic observation of cell morphology and proliferation was performed on cells being cultured.

## Results

Phase composition analysis of the developed cements, carried out 4 weeks after setting and hardening is shown in Table 2. In the case of A1 and A2 materials the presence of two main phases: hydroxyapatite and struvite has been revealed. Analysing the A2 composition—small amounts of calcium sulfate dihydrate (CSD), CaSO_4_·2H_2_O (7 wt%) and brushite—CaHPO_4_·2H_2_O (8 wt%) were detected. In the material B1 also two phases were discovered, i.e. hydroxyapatite and CSD (66 wt%). On the contrary, materials C1 and C2, after setting and hardening, contained only one phase i.e. hydroxyapatite, which was the final product of hydrolysis reaction of α-TCP. The presence of hydroxyapatite as the only crystalline phase in C1 and C2 indicates the high reactivity of the initial α-TCP powder. R1 consisted of hydroxyapatite as the final product of setting reaction, whereas for R2 CSD was formed.

**Table 2:** Phase composition of studied biomaterials after 4 weeks of setting and hardening

Biomaterial	HA (wt%)	Struvite (NH_4_MgPO_4_·6H_2_O) (wt%)	CSD (CaSO_4_·2H_2_O) (wt%)	Brushite (CaHPO_4_·2H_2_O) (wt%)
A1	47	53	–	–
A2	40	45	7	8
B1	34	–	66	–
C1	100	–	–	–
C2	100	–	–	–

The newly developed ceramic materials differed also in open porosity, which ranged from 18 % (for A1) to 46 % (for B1) (Table 3). The obtained potential bone substitutes revealed bimodal pore size distribution with pore diameter from 6 nm to 1.6 µm. Materials C1 and C2 possessed pores with the lowest pore diameters (below 0.48 µm), whereas the biggest voids, ranging from 0.530 to 1.6 µm, were present in cement R1. The observed differences in the open porosity of the obtained materials can influence their sorptive properties and process of interaction with ions present in the culture medium.

**Table 3:** Open porosity and pore size distribution of examined ceramic materials

Biomaterial	Open porosity [%]	Porediameter [µm]
R1	28	0.017–1.600 (I max.: 0.026, II max.: 0.950)
A1	18	0.006–0.760 (I max.: 0.016, II max.: 0.260)
A2	31	0.008–1.200 (I max.: 0.014, II max.: 0.440)
B1	46	0.008–0.980 (I max.: 0.013, II max.: 0.710)
C1	39	0.006–0.480 (I max.: 0.030, II max.: 0.220)
C2	40	0.006–0.470 (I max.: 0.030, II max.: 0.160)

**Fig. 1:**
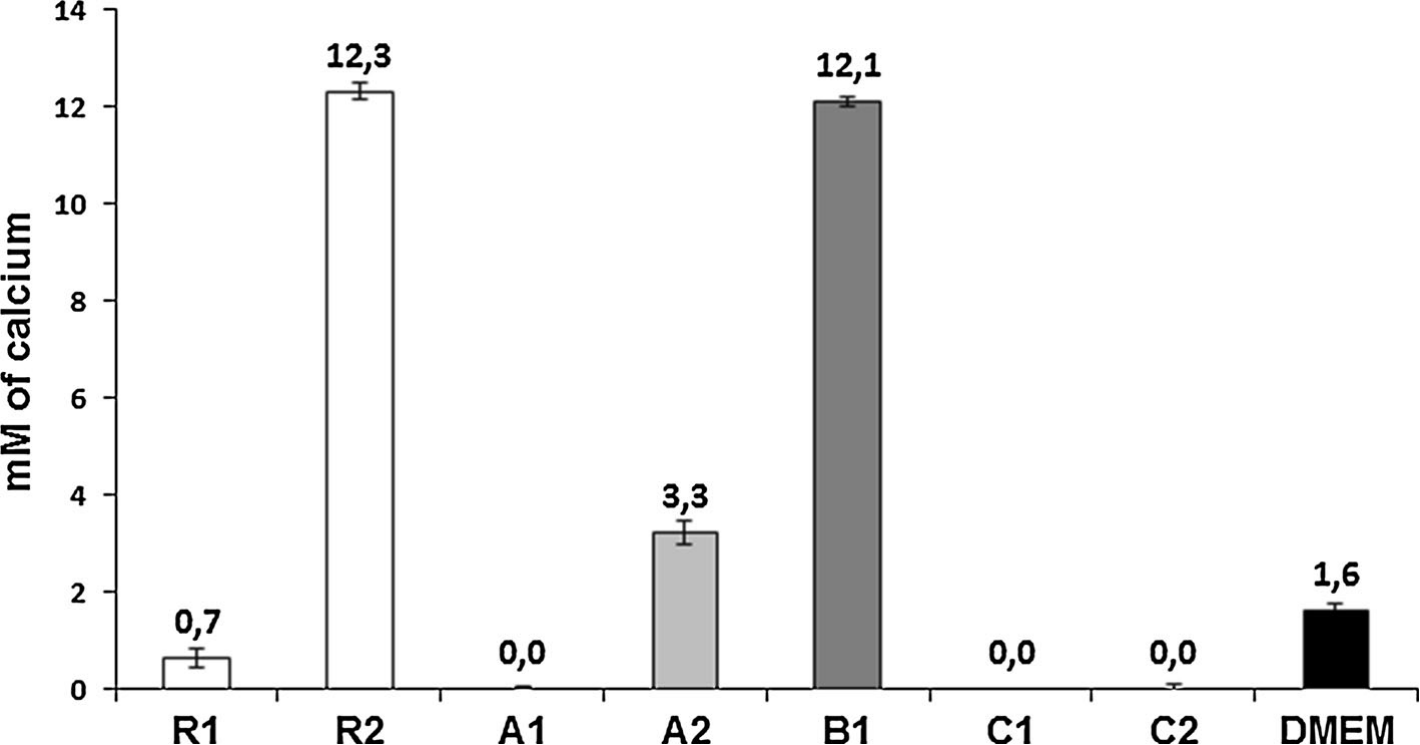
Ca^2+^ concentration in tested extracts, compared to the Ca^2+^ concentration in basal, serum-free culture medium (DMEM)

As shown in Fig. 1, the tested bioceramics influenced the calcium ion concentration in culture medium in various ways. Comparing to the Ca^2+^ concentration in DMEM, which was equal to 1.6 mM, extracts made from R2 and B1 materials contained much higher, i.e. 12.3 mM and 12.1 mM Ca^2+^ concentration, respectively. A2 ceramic also increased the concentration of calcium ions in DMEM, although to a lesser extent. The materials mentioned above underwent the most intensive degradation during the extraction. The other evaluated materials, i.e. A1, C1, C2 and R1 exerted different properties—during 24-h incubation in DMEM the concentration of calcium in extracts was decreased.

**Fig. 2:**
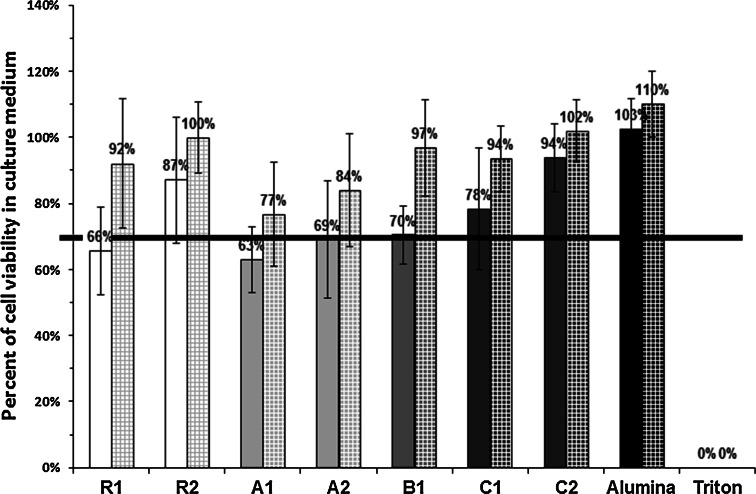
Metabolic activity of hBDC cultured in extracts made of the investigated materials. Values expressed as percent of viability of cells cultured in standard medium (mean ± standard deviation). *Solid bars* represent viability of cells cultured in 100 % extracts, *checked bars* show viability of cells in 50 % extracts. Border cytotoxicity value (70 % of cell viability in standard culture medium) is shown as *horizontal line*. There were no statistically significant differences between the results obtained for the investigated materials and the border cytotoxicity value, except for the R2 and C2, where significantly higher cell viability was found in 50 % extracts (*P* ≪ 0.05 and *P* < 0.01 respectively)

**Fig. 3:**
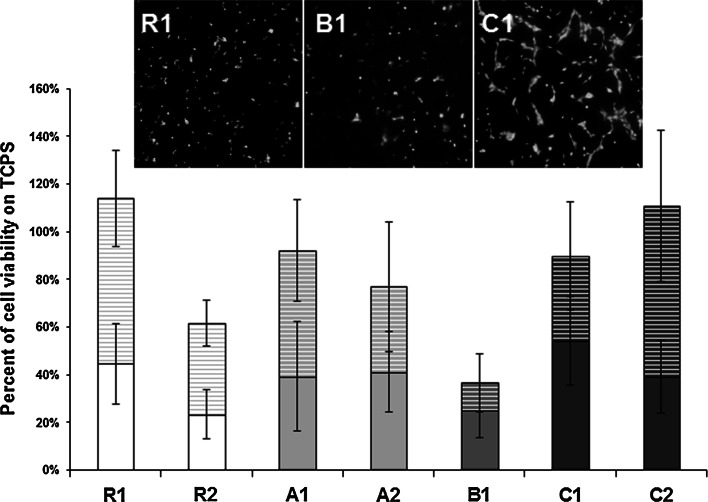
Metabolic activity of human bone-derived cells cultured on the surface of ceramic materials in 24-well culture plate. Values expressed as percent of viability of cells cultured on TCPS (mean ± standard deviation). *Solid bars* represent viability of cells cultured directly on the ceramic samples, while striped bars show viability of cells cultured on TCPS, next to the samples. For the examined materials the aggregate value (cell viability on the material + cell viability next to the material) did not differ significantly from the TCPS control, except for the R2 and B1, where it is significantly lower (*P* < 0.05 and *P* < 0.001 respectively). Microscopic pictures illustrating three types of cell behavior noticed on the surface of the materials (examples comes from the materials: R1, B1, C1) are shown above the chart

**Fig. 4:**
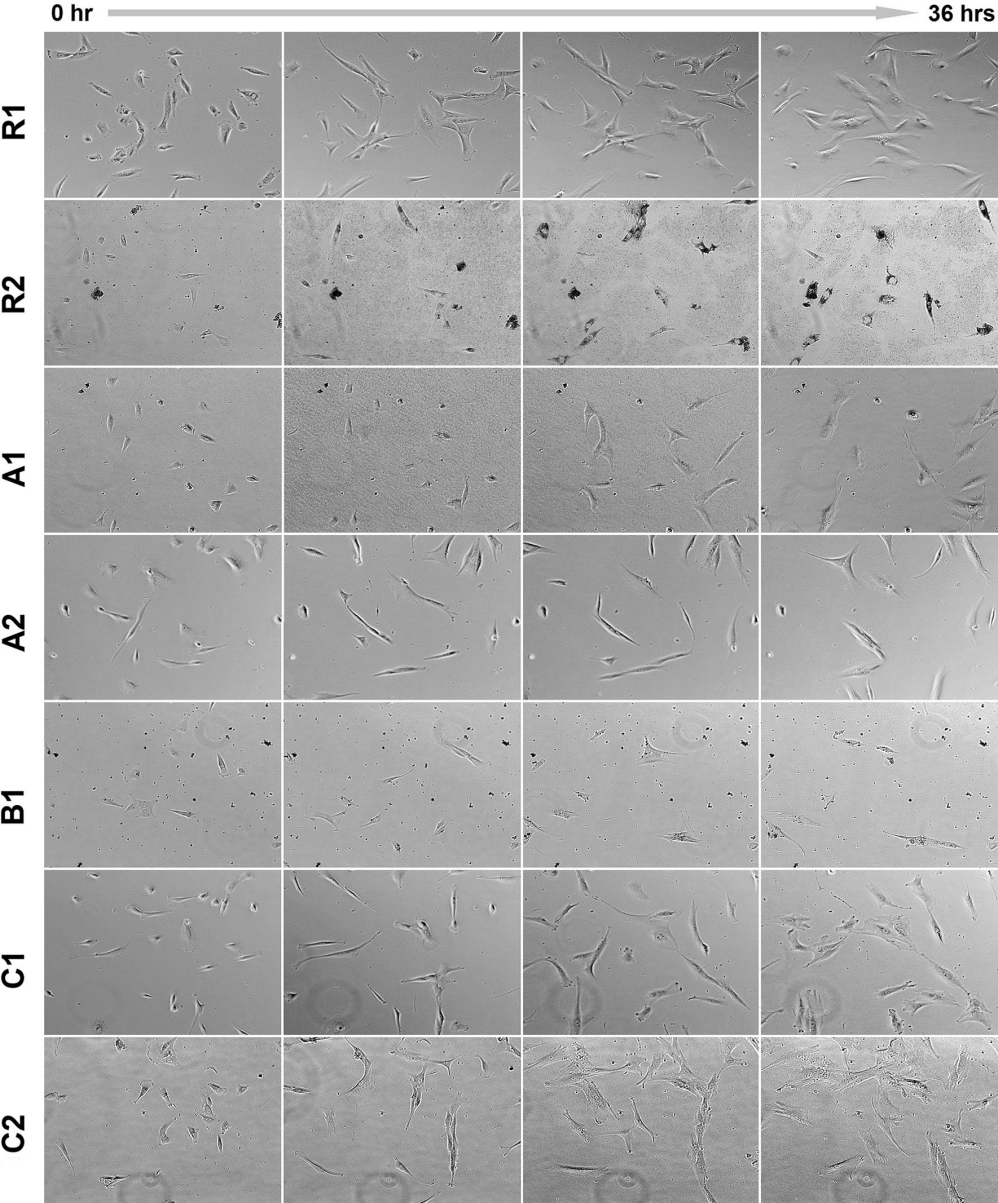
Human bone-derived cells cultured for 36 h next to the investigated materials monitored by time-lapse microscopy. Cell number growing in time can be observed for R1, C1 and C2 ceramics

**Fig. 5:**
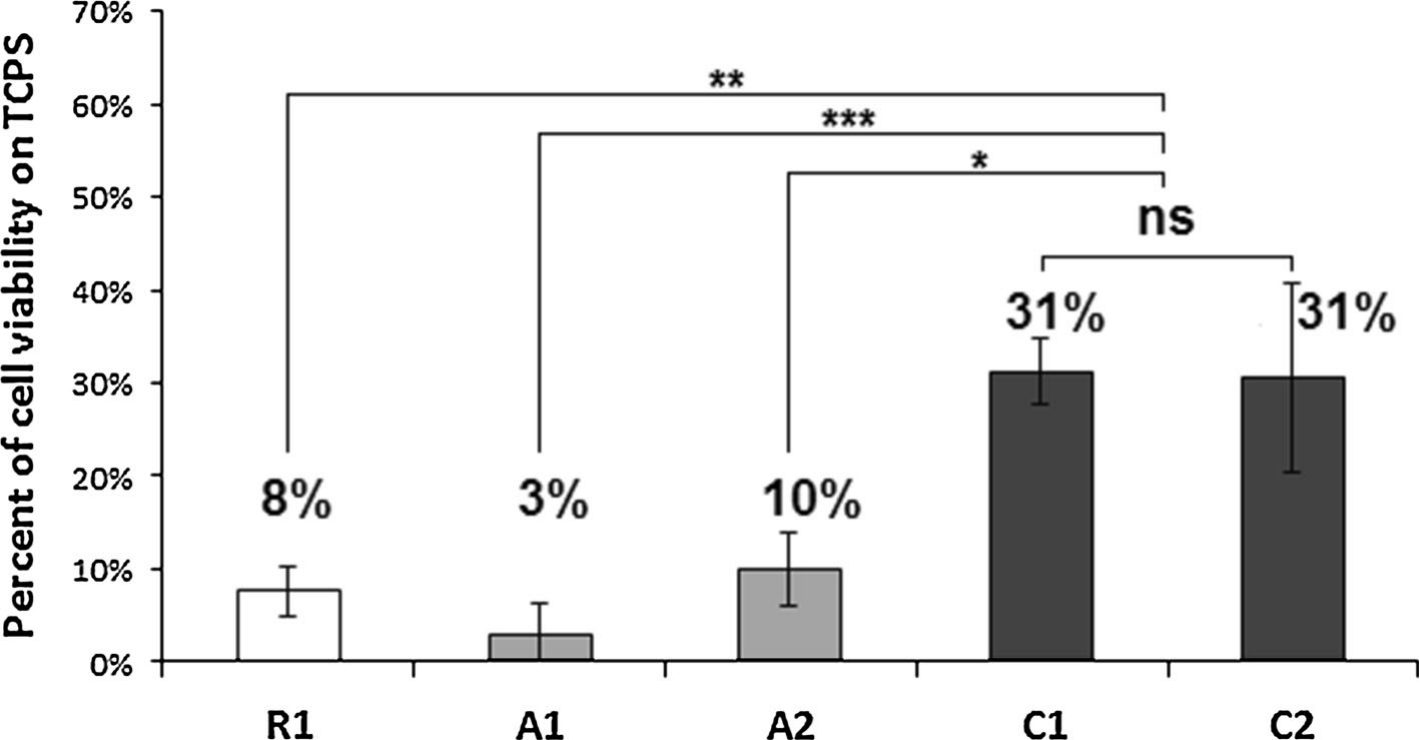
Metabolic activity of cells cultured directly on the surface of ceramic materials in 96-well culture plate. Values expressed as percent of viability of cells cultured on TCPS (mean ± standard deviation). The highest values were obtained for C1 and C2 (no significant differences between them), while the results for the other materials were significantly lower (*P < 0.05; **P < 0.01; ***P < 0.001)

The data presenting cell response to the tested materials are depicted in Figs. 2, 3, 4 and 5. In order to facilitate the tracking of the results, on all the quantitative diagrams the commercial reference materials (R1 and R2) are shown as white bars, HA-based cements are represented by light gray bars, while α-TCP-based ones—by dark gray bars. In the standard trial in extracts, results obtained for alumina and Triton X-100 which were used as the positive and negative controls, are shown in black.

As determined by XTT assay, none of the investigated materials was cytotoxic in the standard trial in extracts. In all trials cell viability was higher or equal to 70 % which was reached in standard culture medium and consider acceptable cytotoxicity level. Therefore, although cell viability was diminished for 100 % (but not in 50 %) extracts from R1, A1, A2, all the tested ceramics successfully passed indirect cytotoxicity assay. Qualitative observation showed numerous cells with regular morphology in the extracts from all the materials regardless of the concentration– there were no differences compared to the control.

For cells cultured in direct contact with the ceramic materials in 24-well plates, part of the population adhered to the bottom of wells, in the close proximity of the sample material. Live/Dead staining revealed the presence of both, the living and dead cells on the surface of the materials. Three types of situations were found: on the surface of C1 and C2 cells were numerous, alive and spread on the material’s surface (example shown for the C1 in Fig. 3), on R1, adhesion of the alive but not spread cells was confirmed (R1 in Fig. 3), while on R2, A1, A2 and B1, mixed population of the dead and live but non-spread cells was observed (example shown for the B1 in Fig. 3). In Alamar Blue assay total metabolic activity of all cells (i.e. cells which adhered to the samples and cells spread on the bottom of wells) was similar to the control in R1, A1, A2, C1, C2 ceramics (Fig. 3). Cells growing on the other ceramics had viability distinctly lower than the control (Fig. 3).

Ceramic materials which showed the highest cytocompatibility in 24-well plate culture were also subjected to observation in more demanding conditions, i.e. in smaller wells where the effect of the tested inserts on the composition of cell culture medium would be more pronounced. The highest survival rate of cells grown in 96-well plates was found for populations seeded on the C1 and C2 (Fig. 5). However, even in these two cases, the metabolic activity of the cells did not exceed 31 % of the activity of the cells cultured on TCPS. For other materials, cell viability was lower than 10 % of control.

Real-time microscopic observation of hBDC cultured next to ceramic samples in 24-well plate showed that cells adhered to the bottom of wells, migrated, proliferated and maintained the regular morphology. Ceramic C2 was the most cytocompatible in this observation. Ceramic particles which were released from the samples of R2, A1, A2 and B1 reduced the growth of cell population (Fig. 4). The most spectacular examples of phagocytosis of the particles accompanying the most intensive release of the ceramic dust were visible in the culture with the R2 material. However, diminished cell motility and increased number of mitoses were observed for all those materials.

## Discussion

The data obtained in our study revealed the substantial differences in the osteogenic cells response towards various CPC, both developed in the laboratory and commercially available. Albeit all the investigated materials have passed a standard assay on cellular extracts successfully, cell behavior in a direct contact with the materials, varied. Two different experimental systems were applied. First, in the culture performed in 24-well culture there are two possibilities for cells to adhere—either directly on the materials’ surface or on the bottom of the culture dish next to the samples (sample surface accounted for about 15 % of the total available area, i.e. 0.28 vs 1.9 cm^2^). In the 96-well plates, the entire surface available for cells was composed of the tested material. Then, the volume of the culture medium was different, i.e. in the 24-well plates the relation of the medium volume to the sample volume was equal to 9 (1 vs 0.11 cm^3^), while in the 96-well plates, the ratio accounted for 1.8 (0.2 vs 0.11 cm^3^). The latter seems to be more advantageous, especially in the systems in which calcium ions release was observed. This was the case for the samples R2, B1 and, to the less extend for A2 (Fig. 1).

The increase in the concentration of calcium ions in the culture medium, found in R2 and B1 materials, is probably due to the presence of CSD in their phase composition (Table 2). Calcium sulfate is a fast resorbable phase and during dissolution substantial amount of Ca^2+^ ions is released into the surrounding environment. It is known that the enhanced Ca^2+^ concentration in culture medium may result in cell death [39–41], therefore we postulate that this process was the main reason for the poor cell viability in the contact with R2 and B1 in vitro. Additionally, materials degradation resulted in releasing of the ceramic particles which were phagocytized by cells. The most massive cellular uptake of the ceramic debris was observed for R2 (Fig. 4). Such phenomenon, if significant, leads to cell death as well. Appearance of ceramic debris may be an important disadvantage in clinical applications. The production of proinflammatory cytokines and markers of osteoclastogenesis by human peripheral blood mononuclear cells in vitro cultured with ceramic particles were reported [42]. The drawbacks related to presence of wear particles have been widely reported in aseptic loosening of orthopedic endoprosthesis [43, 44]. Instability of ceramic cements resulting in releasing of ceramic microparticles might provoke osteolysis and as such may be also harmful in the contact with host tissues in vivo [45].

The moderate enhancement of Ca^2+^ concentration observed for A2 did not influence cells significantly under less demanding culture conditions (large medium volume and culture dish surface availability), while in the 96-well culture, it resulted in as low cell viability as 10 % of the control in average (Fig. 4). Taking these observations together, it can be stated that in case of A2, increase in relation volume of the medium to volume of the sample, as applied in our observations, was good enough to protect cells from the negative consequences of the material degradation in vitro. Therefore, it can be assumed, that it will not generate a problem in the physiological environment in vivo. It is postulated, that A2 may successfully serve as a bone filler. Especially, that the both A-materials have another important advantages, namely, in the composition of set and hardened A1 and A2 materials, there is no adverse, residual MgO which might be potentially cytotoxic; temperature and time of setting process of MPC is controlled due to presence of CSH and sodium pyrophosphate applied as the retarders; and last but not least—multistep resorption is obtained due to differences in physicochemical properties of struvite and hydroxyapatite, in particular their solubility and reactivity. On the other hand, on the basis of our results, the A-materials cannot be postulated as a support for cell delivery, due to the poor cell reaction in the direct contact with its surface. Cells behavior in contact with A1 was similar to A2 in all performed observations. In both cases this is struvite, roughly half and half with hydroxyapatite in the phase composition. Struvite was reported to promote osteoblasts proliferation in comparison to brushite and calcium—deficient HA, but it was not referred to the standard culture dish. Cell spreading was not addressed in this observation while bone-specific markers determined by Western blot were diminished [46]. Not only phase composition but also surface structure may influence capabilities to cell flattening. In our study, it might have been a benefit in the C group.

Definitely, in terms of cytocompatibility, the most favorable materials from the tested groups were cements C1 and C2. They were produced from α-TCP powder, which exhibits high reactivity and hydrolyzes rapidly [12]. Biocompatible hydroxyapatite, similar to the bone apatite, was the only product of setting reaction of cements C1 and C2. In vitro investigations by Czechowska et al. demonstrated that bone cements based on α-TCP did not change significantly pH of simulated body fluid, value of which remained close to the physiological one [12]. As found in our study, cell toleration toward these materials was comparable to the reference R1 in the experimental system based on 24-well culture, i.e. in a relatively high volume of culture medium and the culture surface fulfilled only partly by the investigated materials (Fig. 3). When the entire surface available for cells consisted of the cement, cell viability on the α-TCP-based cements exceeded the value obtained for R1 about three times (Fig. 5). Interestingly, a remarkable reduction of the Ca^2+^ concentration in the medium observed for C1 and C2 (see Fig. 1), did not affect cell spreading, although such effect might have been expected in view of the poor cell spreading reported on the surface of calcium-deficient hydroxyapatite, which diminished Ca^2+^ concentration in medium [46]. In the case of C1 and C2 the reduction of Ca^2+^ in medium might be caused by the presence of polymers introduced with cement liquids. Chitosan is well known for its chelating properties, which may cause changes in concentration of selected ions in aqueous surroundings. Sorption of calcium ions may be also provoked by the microstructure of hardened cement bodies. Presence of small capillary pores favors the sorption process of ions from the environment. On the other hand, the presence of small pores on the surface of these materials may be of great advantage. In the experiment of Park et al. where cells in culture were located on the vertically oriented TiO_2_ nanotubes with defined diameters between 15 and 100 nm, significant differences in cell attachment and spreading were reported [47]. Cell adhesion and spreading were excellent, on the tubes of 15 nm in diameter, while a spacing larger than 50 nm resulted in fail of cell attachment and cell death. Due to the convincing explanation postulated by the authors, cell spreading was dependent on the availability of the support for focal contact organization. Therefore, among the other obvious factors influencing the cell-material reaction, also this element may play a role. In our study, for C1 and C2, pore diameter is the smallest of the tested materials (see Table 3), thus the frequency with which the cell meets place to organize focal contact is the highest.

Due to the classical definition, biocompatibility is understood as “the ability of a material to perform with an appropriate host response in a specific application” [48]. Therefore, among the cements investigated in our study, only α-TCP-based CPC, which give the best support for cells in a direct contact, may be taken into account for cell-donor systems to be applied in regenerative medicine. Positive results from cell-based in vitro observations along with good materials characteristics, such as excellent cohesion and handling properties, make cements on the basis of α-TCP materials potentially superior to the other cement materials currently used in clinical practice.

## Conclusions

The in vitro evaluation of biocompatibility of the cement type implant materials composed of calcium and magnesium phosphates and CSH assessed on the basis of cell cultures, revealed that the results strongly depend on cell culture methods. The experiments which were performed with cells growing directly on the surface of the investigated materials resulted in significantly different outcomes, when compared to the indirect approach. The ratio of culture medium volume to the size of the tested inserts was found to be crucial if release or uptake of calcium ions from the materials occurred.

The materials consisting of CSH, which is a fast degrading component—commercial Surgi Plaster™ (R2) and newly developed B1 and A2—had negative impact on the cells. These materials not only introduce a huge amount of calcium ions but also may release CSD particles, afterward internalized by cells. The remaining materials i.e. commercial HydroSet™ (R1) as well as the developed bone substitutes (α-TCP-based C1, C2, and magnesium phosphate A1 cement) did not have negative impact on the cells except the experiments with the smallest medium volume. Substantial decrease of calcium ions concentration in the culture medium may be the explanation to this phenomenon. Since this effect was successfully compensated by excess volume of medium, it is likely to be eliminated when applied to host tissues in vivo. On the basis of the cytocompatibility studies, the most recommended among the developed bone substitutes were the α-TCP-based materials: C2 and C1. Generally, chemically bonded ceramics were found to be less biologically stable in comparison to the sintered ceramic materials based on calcium phosphates and CSH.

The obtained results strongly support the need to run a series of tests for cement type materials, which significantly change concentration of calcium ions in the culture media. Even though, in vitro studies may not be representative for the host tissue tolerance and response to materials, they still show the differences between the materials. Moreover, at the screening stage they facilitate the decision which materials from the broad offer can be taken into account for further preclinical observations.

## References

[1] Chen JC, Ko CL, Shih CJ, Tien YC, Chen WC (2012). Calcium phosphate bone cement with 10 wt% platelet-rich plasma in vitro and in vivo. J Dent.

[2] LeGeros RZ (1993). Biodegradation and bioresorption of calcium phosphate ceramics. Clin Mater..

[3] Schmitz JP, Hollinger JO, Milam SB (1999). Reconstruction of bone using calcium phosphate bone cements: a critical review. J Oral Maxillofac Surg.

[4] Zhang JT, Tancret F, Bouler JM (2011). Fabrication and mechanical properties of calcium phosphate cements (CPC) for bone substitution. Mater Sci Eng C.

[5] El-Ghannam A, Ducheyne P, Ducheyne P (2011). Bioactive ceramics. Comprehensive biomaterials.

[6] Apelt D, Theiss F, El-Warrak AO, Zlinszky K, Bettschart-Wolfisberger R, Bohner M (2004). In vivo behavior of three different injectable hydraulic calcium phosphate cements. Biomaterials.

[7] Arisan V, Ozdemir T, Anil A, Jansen JA, Ozer K (2008). Injectable calcium phosphate cement as a bone-graft material around peri-implant dehiscence defects: a dog study. Int J Oral Maxillofac Implants.

[8] Ginebra MP, Traykova T, Planell JA (2006). Calcium phosphate cements as bone drug delivery systems: a review. J Control Release.

[9] Lee GS, Park JH, Shin US, Kim HW (2011). Direct deposited porous scaffolds of calcium phosphate cement with alginate for drug delivery and bone tissue engineering. Acta Biomater.

[10] Moreau JL, Xu HHK (2009). Mesenchymal stem cell proliferation and differentiation on an injectable calcium phosphate—chitosan composite scaffold. Biomaterials.

[11] Carrodeguas RG, De Aza S (2011). alpha-Tricalcium phosphate: synthesis, properties and biomedical applications. Acta Biomater.

[12] Czechowska J, Zima A, Paszkiewicz Z, Lis J, Slosarczyk A (2014). Physicochemical properties and biomimetic behaviour of α-TCP-chitosan based materials. Ceram Int.

[13] Lilley KJ, Gbureck U, Knowles JC, Farrar DF, Barralet JE (2005). Cement from magnesium substituted hydroxyapatite. J Mater Sci Mater Med.

[14] Zima A, Paszkiewicz Z, Siek D, Czechowska J, Ślósarczyk A (2012). Study on the new bone cement based on calcium sulfate and Mg, CO_3_ doped hydroxyapatite. Ceram Int.

[15] Alkhraisat MH, Rueda C, Marino FT, Torres J, Jerez LB, Gbureck U (2009). The effect of hyaluronic acid on brushite cement cohesion. Acta Biomater.

[16] Deb S (2008). Orthopaedic bone cements.

[17] Komath M, Varma HK (2003). Development of a fully injectable calcium phosphate cement for orthopedic and dental applications. Bull Mater Sci.

[18] Mestres G, Ginebra MP (2011). Novel magnesium phosphate cements with high early strength and antibacterial properties. Acta Biomater.

[19] Soudee E, Pera J (2000). Mechanism of setting reaction in magnesia-phosphate cements. Cem Concr Res.

[20] Yu YL, Wang J, Liu CS, Zhang BW, Chen HH, Guo H (2010). Evaluation of inherent toxicology and biocompatibility of magnesium phosphate bone cement. Colloids Surf B Biointerfaces.

[21] Tamimi F, Le Nihouannen D, Bassett DC, Ibasco S, Gbureck U, Knowles J (2011). Biocompatibility of magnesium phosphate minerals and their stability under physiological conditions. Acta Biomater.

[22] Pijocha D, Łój G, Nocuń-Wczelik W, Ślósarczyk A (2011). Effect of retardants on the heat release during setting of bone cement-type composites. J Achiev Mater Manuf Eng..

[23] Pijocha D, Zima A, Paszkiewicz Z, Slosarczyk A (2013). Physicochemical properties of the novel biphasic hydroxyapatite-magnesium phosphate biomaterial. Acta Bioeng Biomech..

[24] Garg AK, Garg AK (2004). Review of bone-grafting materials. Bone biology, harvesting and grafting for dental implants: rationale and clinical applications.

[25] Knabe C, Houshmand A, Berger G, Ducheyne P, Gildenhaar R, Kranz I (2008). Effect of rapidly resorbable bone substitute materials on the temporal expression of the osteoblastic phenotype in vitro. J Biomed Mater Res A.

[26] Jung HM, Song GA, Lee YK, Baek JH, Ryoo HM, Kim GS (2010). Modulation of the resorption and osteoconductivity of alpha-calcium sulfate by histone deacetylase inhibitors. Biomaterials.

[27] Mamidwar S, Weiner M, Alexander H, Ricci J (2008). In vivo bone response to calcium sulfate/poly l-lactic acid composite. Implant Dent..

[28] Thomas MV, Puleo DA (2009). Calcium sulfate: properties and clinical applications. J Biomed Mater Res B Appl Biomater..

[29] Bahn SL (1966). Plaster—a bone substitute. Oral Surg Oral Med Oral Pathol Oral Radiol Endod.

[30] Nilsson M, Wang JS, Wielanek L, Tanner KE, Lidgren L (2004). Biodegradation and biocompatibility of a calcium sulphate-hydroxyapatite bone substitute. J Bone Joint Surg Br.

[31] Zhang JT, Liu WZ, Schnitzler V, Tancret F, Bouler JM (2014). Calcium phosphate cements for bone substitution: chemistry, handling and mechanical properties. Acta Biomater.

[32] Gallagher JA, Gundle R, Beresford JN (1996). Isolation and culture of bone-forming cells (osteoblasts) from human bone. Methods Mol Med.

[33] Kudelska-Mazur D, Lewandowska-Szumiel M, Mazur M, Komender J (2005). Osteogenic cell contact with biomaterials influences phenotype expression. Cell Tissue Bank..

[34] Scudiero DA, Shoemaker RH, Paull KD, Monks A, Tierney S, Nofziger TH (1988). Evaluation of a soluble tetrazolium/formazan assay for cell growth and drug sensitivity in culture using human and other tumor cell lines. Cancer Res.

[35] Schreer A, Tinson C, Sherry JP, Schirmer K (2005). Application of Alamar blue/5-carboxyfluorescein diacetate acetoxymethyl ester as a noninvasive cell viability assay in primary hepatocytes from rainbow trout. Anal Biochem.

[36] Willerth SM, Arendas KJ, Gottlieb DI, Sakiyama-Elbert SE (2006). Optimization of fibrin scaffolds for differentiation of murine embryonic stem cells into neural lineage cells. Biomaterials.

[37] ISO. Biological evaluation of medical devices. Part 12: sample preparation and reference materials. Geneva: International Organization for Standardization (ISO); 2007.

[38] ISO. Biological evaluation of medical devices. Part 5: tests for in vitro cytotoxicity. Geneva: International Organization for Standardization (ISO); 2009.

[39] Adams CS, Mansfield K, Perlot RL, Shapiro IM (2001). Matrix regulation of skeletal cell apoptosis—role of calcium and phosphate ions. J Biol Chem.

[40] Malafaya PB, Reis RL (2009). Bilayered chitosan-based scaffolds for osteochondral tissue engineering: influence of hydroxyapatite on in vitro cytotoxicity and dynamic bioactivity studies in a specific double-chamber bioreactor. Acta Biomater.

[41] Tsang EJ, Arakawa CK, Zuk PA, Wu BM (2011). Osteoblast interactions within a biomimetic apatite microenvironment. Ann Biomed Eng.

[42] Velard F, Braux J, Amedee J, Laquerriere P (2013). Inflammatory cell response to calcium phosphate biomaterial particles: an overview. Acta Biomater.

[43] Goodman SB, Ma T, Chiu R, Ramachandran R, Smith RL (2006). Effects of orthopaedic wear particles on osteoprogenitor cells. Biomaterials.

[44] Greenfield EM, Bi Y, Ragab AA, Goldberg VM, Van De Motter RR (2002). The role of osteoclast differentiation in aseptic loosening. J Orthop Res.

[45] Ambard AJ, Mueninghoff L (2006). Calcium phosphate cement: review of mechanical and biological properties. J Prosthodont..

[46] Ewald A, Helmschrott K, Knebl G, Mehrban N, Grover LM, Gbureck U (2011). Effect of cold-setting calcium- and magnesium phosphate matrices on protein expression in osteoblastic cells. J Biomed Mater Res B Appl Biomater.

[47] Park J, Bauer S, von der Mark K, Schmuki P (2007). Nanosize and vitality: TiO_2_ nanotube diameter directs cell fate. Nano Lett.

[48] Williams DF, European Society for Biomaterials. Definitions in biomaterials. Progress in biomedical engineering, vol 4. In: Proceedings of a consensus conference of the European Society for Biomaterials, Chester, March 3–5, 1986. New York: Elsevier; 1987

[49] Olkowski R, Kaszczewski P, Lewandowska-Szumieł M, Czechowska J, Siek D, Pijocha D (2012). Conditions of in vitro culture influence the viability of human bone cells cultured with ceramic materials. Eng Biomater..

